# Accuracy of HVL measurements utilizing solid state detectors for radiography and fluoroscopy X‐ray systems

**DOI:** 10.1002/acm2.13389

**Published:** 2021-08-10

**Authors:** Pei‐Jan P. Lin, Allen R. Goode

**Affiliations:** ^1^ Department of Radiology Virginia Commonwealth University Medical Center Richmond VA USA; ^2^ Department of Radiology and Medical Imaging University of Virginia Health System Charlottesville VA USA

**Keywords:** accuracy of HVL estimated, calculated HVL, consistency of HVL

## Abstract

The half‐value layer (HVL) is one of the regulatory required radiation safety parameters that needs to be measured annually. With the advent of solid state detectors and their associated electrometer assembly, the HVL measurement can be conducted with relative ease. In fact, various radiological technique parameters such as tube potential (kV), exposure time in millisecond (msec), air kerma (mGy), and air kerma rate (mGy/sec) can be obtained along with the HVL with just one exposure. The measured (or, calculated) HVL is based on radiation detection systems calibrated for conventional x‐ray systems equipped with tungsten anode and added aluminum filters (molybdenum anode and filter in the case of mammography systems). However, a new generation of radiography and fluoroscopy (R/F) systems, inclusive of interventional angiography equipment, is equipped with varying thicknesses and materials of spectral shaping filters (SSF) to minimize the radiation exposure to the patients while image quality is maintained and optimized. The accuracy of HVL obtained with new generation of R/F systems has not been investigated in depth due to the addition of spectral filters yielding a harder beam quality with a higher HVL than the regulatory required value of 2.9 mm Al HVL at 80 kV. It would be of great interest to determine the accuracy of HVL as measured (or, calculated) by the solid state detector systems (SSDS), especially when accurate radiation dose delivered to the patient is required. In this investigation, the subject is limited to the accuracy of HVL measurement for conventional R/F systems.

## 1 INTRODUCTION

The minimum required half‐value layer (HVL) as specified by the Food and Drug Administration (FDA) for radiography and fluoroscopy x‐ray tubes is 2.9 millimeters of aluminum (mm Al) at 80 kV (or 1.8 mm Al at 70 kV).^1^ Typically, compliance testing may be measured at 70 kV or 80 kV on an R/F system. New generations of R/F systems are equipped with spectral shaping filters (SSF) such as aluminum (Z = 13), copper (Z = 29), and titanium (Z = 22), which are inserted into the primary beam during clinical operation in addition to the minimum required filtration. Thus, additional SSF inserted into the primary beam increase the beam quality in excess of 2.9 mm Al at 80 kV.

It is well publicized that fluoroscopy systems employ SSFs either dynamically or statically^2^ under the Automatic Brightness Control (ABC), or Automatic Dose Rate Control (ADRC) mode of operation. On the other hand, radiographic equipment has been equipped with SSFs, typically 0.1 or 0.2 millimeter of copper (mm Cu), at least for the past 20 years. For radiographic examinations, different thicknesses of copper filters are preprogrammed with the Automatic Exposure Control (AEC) circuitry in accordance to different parts of anatomy selected for examination. In general, during the annual equipment performance evaluation (EPE), when testing the minimum filtration requirement, the SSF should not be included. This is to assure that the regulatory required minimum filtration is complied with, irrespective of whether the imaging system is a radiographic or a fluoroscopic system.

Unless there is a need to assess the patient skin dose for more accurate patient dosimetry, the annual evaluation of the minimum filtration requirement is considered complete and no further HVL measurements may need to be performed. However, we have noticed the HVL obtained with this one‐shot method lacks some consistency from one year to another and between two different calibration dates of the same solid state detector system (SSDS). Since the HVL measured with a SSDS is calculated and estimated using proprietary hardware and software unique to a given SSDS manufacturer, we have decided to evaluate the accuracy of measured HVL against the HVL measured with the traditional test methodology of narrow beam geometry.^3^ Lastly, since HVL is used in the calculation of Peak Skin Dose, a procedure for which there is a Current Procedural Terminology code (CPT 76145) as of January 2021,^4^ a thorough evaluation of the HVL measurement accuracy is necessary.

## 2 MATERIALS AND METHODS

HVL assessment was performed on a Siemens Axiom Artis Zee (Siemens Healthineers, Forchheim, Germany) interventional angiography system.^5^ The system was tested in service mode, which provided access to manual control of the SSF (0, 0.1, 0.2, 0.3, 0.6, and 0.9 mm Cu). Under service mode, tube current (mA) can be held constant while the operator can select combinations of kV and SSF that would occur under normal clinical operations.

The radiation detection systems used to quantify HVL included four systems that are available to this institution. Since the main theme of this investigation is to determine the accuracy of the “single shot” HVL indicated by the SSDS, manually calculated HVLs determined by Al filters and ionization chamber system were used as the reference. The details of the ionization chamber system and the three SSDS systems are listed as shown in Table 1.

**TABLE 1:** Radiation detection systems employed

	Electrometer	Detector type (Model #)	Calibration accuracy	Software
Radcal	AGT‐P‐AD	Ion chamber (10X6‐10)	Energy Response ±5%, 20 keV to 1.33 MeV	Accu‐gold 2
	Accu‐gold+touch	SSDS (AGMS‐DM+)	HVL (mmAl) ±5% or ±0.06 mmAl Range: 1.3 −13.5 or ±0.2 mmAl, (whichever is greater.)	
RTI	Piranha 657 [Unit 1]	SSDS (Built‐in)	HVL (mmAl) ±10% or ±0.2 mm (60–120 kV, HF/DC, >10 µGy/s)	Ocean 2014
	Piranha 657 [Unit 2]	SSDS (Built‐in)	Range: 1.2–14 mm Al (35–150 kV, TF=1–45 mmAl)*	

^a^ The HVL range is valid if the Total Filtration (TF) is also within the specified range. For high TF at high kV the HVL range may be limited. TF: Total Filtration.

**FIGURE 1:**
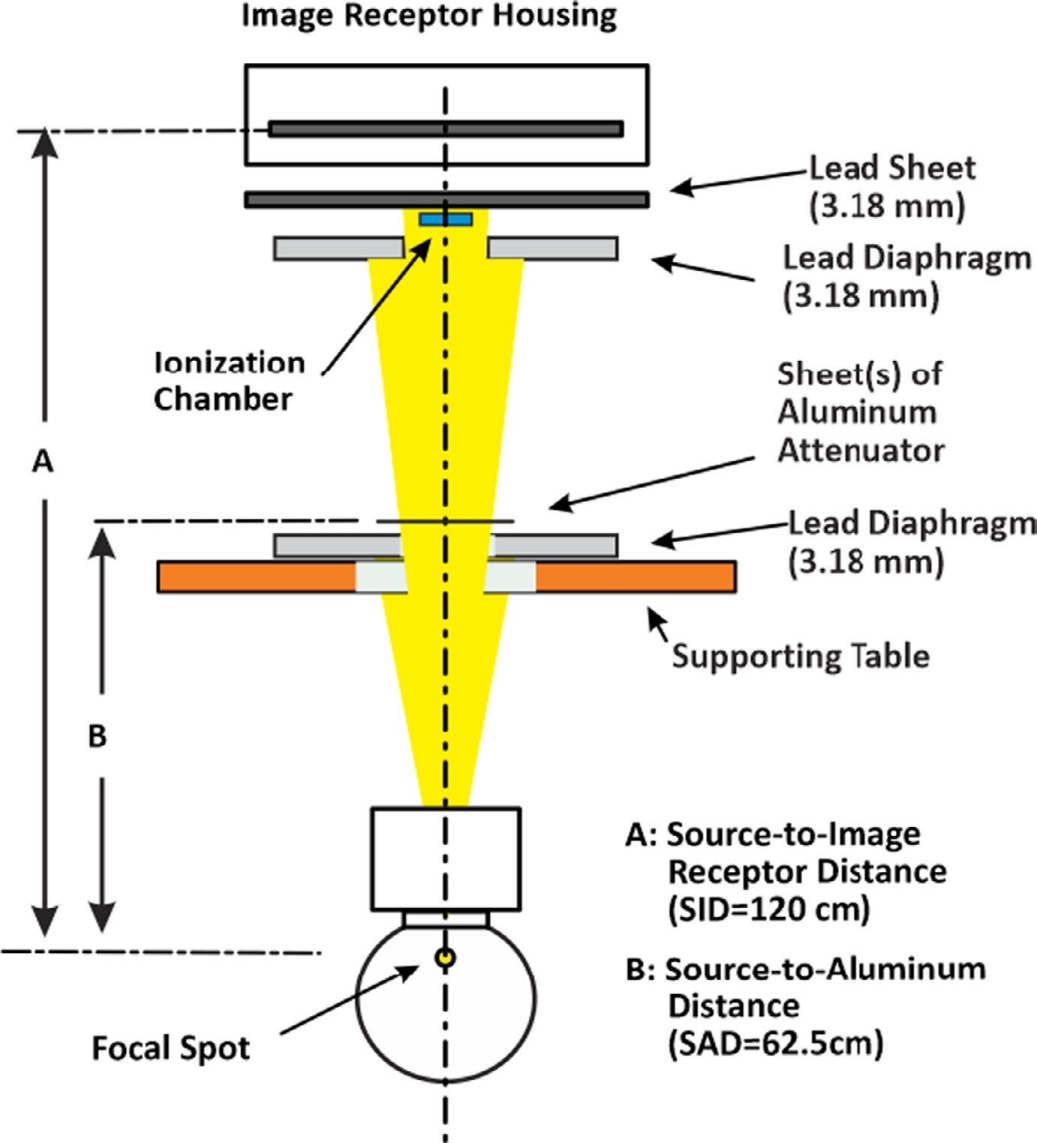
The geometry of HVL measurements utilizing the ionization chamber

Figure 1 illustrates the measurement geometry used for testing. The reference HVL measurements utilizing the ionization chamber were setup in accordance to the geometry set forth by the American Society for Testing and Materials (ASTM) Designation F3094‐14,^6^ with minor modifications for the measurements. Minor modifications were necessary to the setup since the radiation source employed for our experiment was an interventional angiography room installed for clinical applications. The measurement procedure is similar to the narrow beam method commonly seen in physics textbooks for HVL measurements.

For testing, the tube potential was varied from 60 kV to 120 kV in 10 kV increments. For the HVL measurements with SSDS, in place of the aluminum attenuator in Figure 1, the solid state detector is positioned up‐side‐down facing the X‐ray tube. In one single exposure, the SSDS displays the tube potential (kV), air kerma (mGy), exposure time in milliseconds (msec), HVL (mm Al), and total filtration (mm Al). In this study, each HVL measurements were conducted with four exposures to obtain the average value. We are specifically interested in the HVL. However, for comparison among the three (Radcal and two RTI) SSDS units, the tube potential was also monitored to ensure consistency of X‐ray tube potential applied.

## 3 RESULTS

The measurement results are listed in Table 2, which is divided into four sections where section A is for the ionization chamber, sections B and C are for RTI SSDS Units # 1 and # 2 (Model Name Piranha), respectively. The data shown in section D is for the Radcal SSDS (Model Name AGMS‐DM+). The cells (with yellow background) in section B for the 0.9 mm Cu SSF at 60 kV and 70 kV, the values entered are from the same RTI Unit #1 with software/calibration version at least two calibration cycles ago. The blank cells in Table 2, Section C, for the 0.9 mm Cu at the same 60 kV and 70 kV cells are “out‐of‐range” of the Ocean 2014 software (current calibration version in 2019) and no HVL values were displayed at the time of measurements (refer to additional comments on this matter in the discussion section). The values listed in the second row of each section are the tube potential applied for the HVL measurements. We found the applied tube potential is consistent, better than ±0.6 kV, throughout the duration of testing. The accuracy for each kV tested was within ±1.2 kV of the preset values. The first column of Table 2 is the SSF (mm Cu) manually selected from the control console. The HVLs measured are in millimeter of aluminum (mm Al).

**TABLE 2:** The HVL

A		HVL (mm Al) RADCAL Model 10X6‐10 Ion Chamber						
Spectral Shaping Filter	kV→	60	70	80	90	100	110	120
	0 mm Cu	2.2	2.64	3.14	3.5	3.8	4.17	4.61
	0.1 mm Cu	3.54	4.16	4.8	5.45	5.87	6.38	6.94
	0.2 mm Cu	4.37	5.19	5.95	6.68	7.17	7.71	8.27
	0.3 mm Cu	5	5.94	6.77	7.54	8.05	8.63	9.2
	0.6 mm Cu	6.12	7.2	8.17	9.02	9.59	10.2	10.78
	0.9 mm Cu	6.79	8	9.05	9.9	10.54	11.13	11.66
B		HVL (mm Al) RTI Piranha Unit #1						
Spectral Shaping Filter	kV→	60	70	80	90	100	110	120
	0 mm Cu	2.24	2.62	3.04	3.44	3.91	4.38	4.86
	0.1 mm Cu	3.54	4.14	4.73	5.33	5.89	6.43	6.93
	0.2 mm Cu	4.3	5.03	5.73	6.39	6.99	7.55	8.06
	0.3 mm Cu	4.91	5.69	6.44	7.14	7.78	8.33	8.85
	0.6 mm Cu	5.83	6.87	7.77	8.52	9.15	9.7	10.3
	0.9 mm Cu	6.42	7.51	8.47	9.28	9.91	10.5	11.1
C		HVL (mm Al) RTI Piranha Unit #2						
Spectral Shaping Filter	kV→	60	70	80	90	100	110	120
	0 mm Cu	2.22	2.6	3.01	3.41	3.86	4.33	4.8
	0.1 mm Cu	3.5	4.08	4.68	5.28	5.83	6.36	6.87
	0.2 mm Cu	4.27	4.97	5.67	6.33	6.94	7.49	8.01
	0.3 mm Cu	4.84	5.61	6.38	7.08	7.72	8.27	8.79
	0.6 mm Cu	5.79	6.78	7.7	8.47	9.12	9.69	10.3
	0.9 mm Cu			8.48	9.24	9.88	10.5	11.1
D		HVL (mm Al) Radcal AGMS‐DM+						
Spectral Shaping Filter	kV→	60	70	80	90	100	110	120
	0 mm Cu	2.27	2.65	3	3.35	3.7	4.07	4.46
	0.1 mm Cu	3.47	4.1	4.65	5.2	5.73	6.22	6.69
	0.2 mm Cu	4.31	5.11	5.81	6.46	7.05	7.62	8.13
	0.3 mm Cu	4.92	5.82	6.62	7.33	7.97	8.53	9.04
	0.6 mm Cu	5.91	6.99	7.91	8.7	9.36	9.92	10.41
	0.9 mm Cu	6.65	7.85	8.88	9.72	10.42	11.06	11.59

**FIGURE 2:**
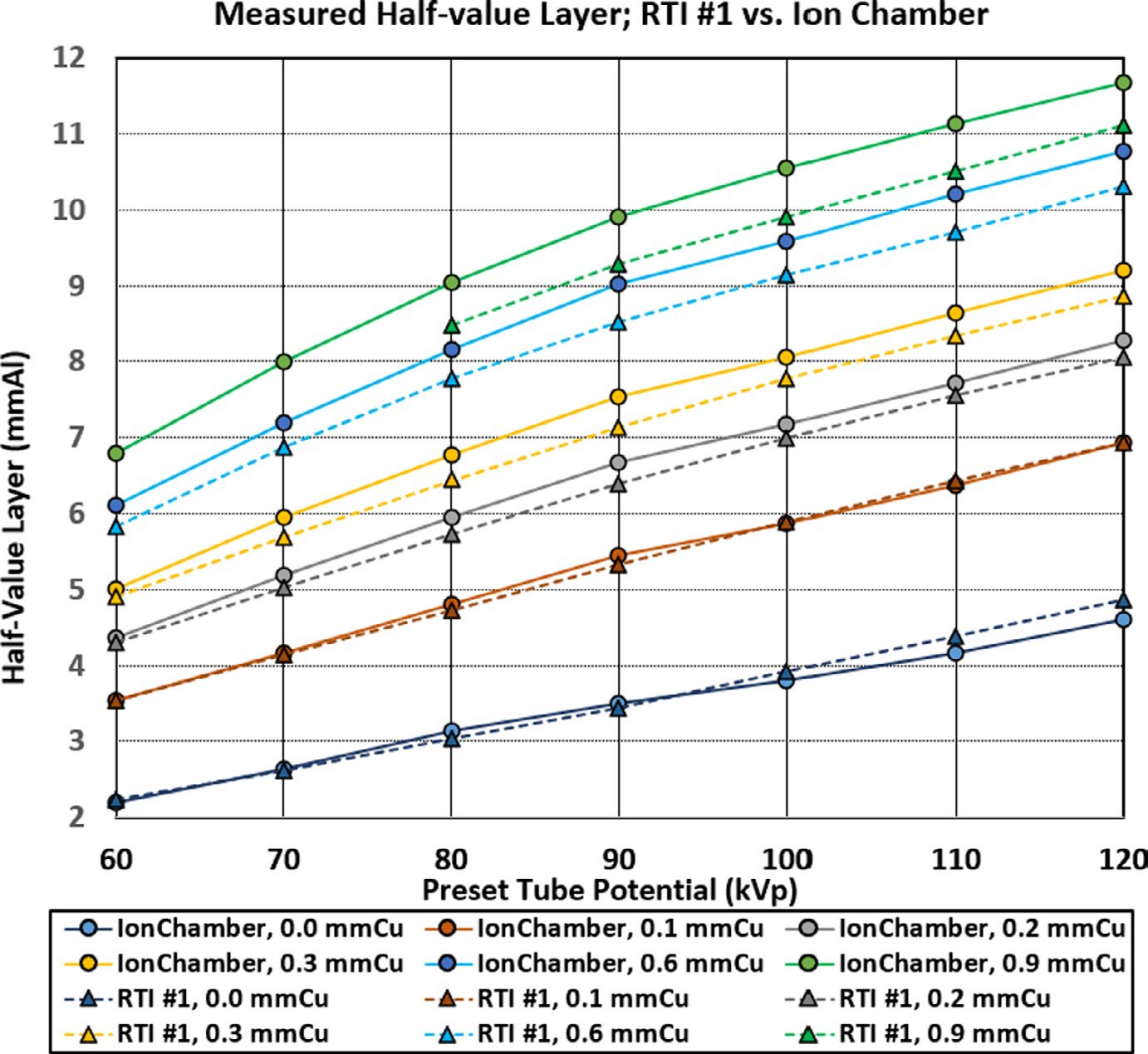
The HVLs for the RTI Unit #1 vs. Ionization Chamber. The measured HVL for RTI #1 SSDS plotted against the Ion Chamber. The higher the tube potential and the thicker the copper SSF, the discrepancy becomes larger. Note that two data points for RTI #1 At 60 kV and 70 kV with SSF of 0.9 mm Cu was supplemented with HVL measured in the past (on the same angiography unit two years earlier) with a different calibration look up table since these two data points were out‐of‐range with the current calibration

First, let us compare the HVLs for RTI Unit #1 with the Radcal ionization chamber. The data for these two detectors is plotted in Figure 2. The solid lines with circles are for the Radcal ionization chamber and the dotted lines with triangles are for the RTI Unit #1.

It is apparent that the measured HVLs are in good agreement when the SSFs are set to 0 mm Cu and 0.1 mm Cu. However, as the SSF is increased (0.2 mm Cu and thicker), the RTI Unit #1 consistently underestimates the HVL. In comparing the HVL results against that of Radcal ionization chamber, the discrepancy in the HVL estimation is +0.40 /−0.56 mm Al over the entire measurement range of spectral shaping filters in combination with the tube potentials (hereafter; SSF‐kV range). For the RTI Unit #2, the discrepancy in the HVL estimation is +0.34 /−0.56 mm Al over entire SSF‐kV range.

**FIGURE 3:**
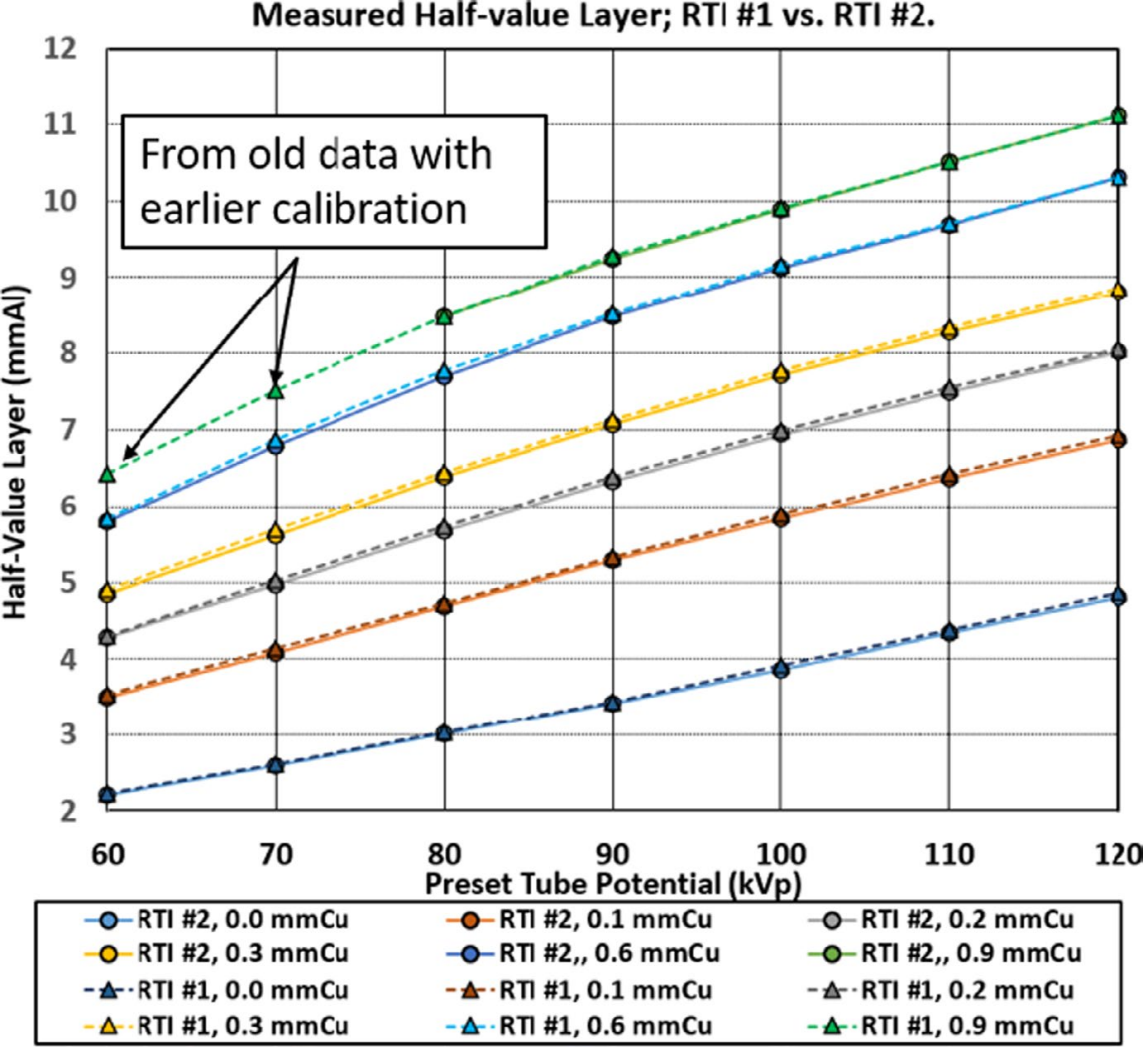
The HVLs for the RTI Unit #1 vs. RTI Unit #2. Comparison of HVL measured between RTI #1 and RTI #2. The curves of each corresponding HVL are almost identical, which indicates the calibration provided to two different RTI SSDS is consistent. Both SSDS at 60 kV and 70 kV were out‐of‐range due to the software update. The corresponding data points for RTI #1 are from previously obtained values

The graphical comparison of the measured HVLs between RTI Units #1 and #2, as depicted in Figure 3, is in a very good agreement of ±0.09 mm Al over entire SSF‐kV range. It is interesting to note that these two units were calibrated approximately one year apart, showing good consistency in the calibration procedure.

**FIGURE 4:**
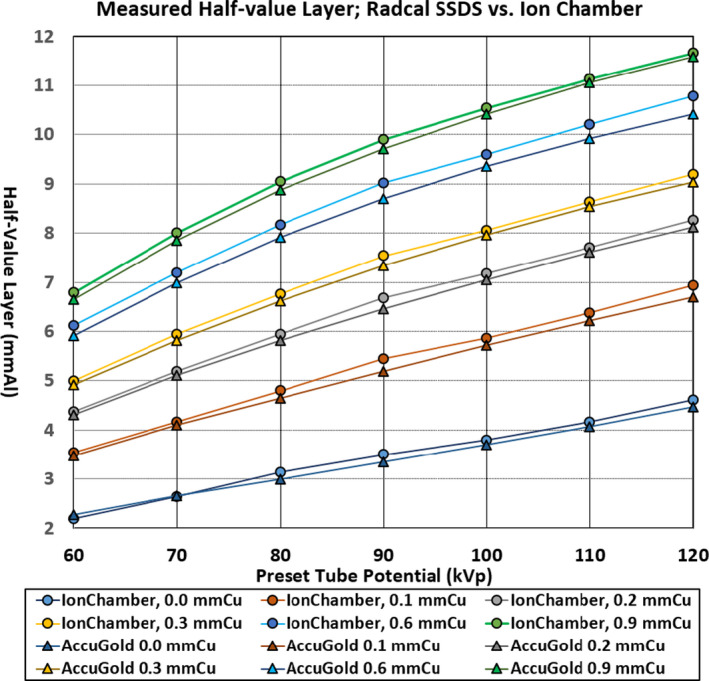
The HVLs for Radcal AccuGold (SSDS) vs. Ionization Chamber. The HVLs measured with Radcal Accu Gold SSDS against the ion chamber. The discrepancy of HVLs measured with Radcal Accu Gold vs. ion chamber is within 0.5 mm Al

Depicted in Figure 4 is graphical comparison of Radcal SSDS HVL against the HVL obtained with the Radcal ionization chamber via calculation. The corresponding SSF curves of both detectors show they are closely following each other for the SSF‐kV range. The measured HVLs using Radcal SSDS appear to have good correction applied to match the Radcal ionization chamber and the discrepancy in the HVL estimation is +0.37 /−0.07 mm Al over entire SSF‐kV range.

It can be said both SSDS detectors manufactured by RTI and Radcal are similar in their calibration accuracy against the ionization chamber. They both have better and more accurate HVL estimation at 0 mm Cu and 0.1 mm Cu SSF compared against SSF of 0.2 mmCu and thicker. The HVL measured by SSDS is accurate, especially, in the low tube potentials (60 kV to 80 kV). However, as the beam quality is increased (with increase in copper filter thickness), the discrepancy in measured HVL becomes larger.

## 4 DISCUSSION AND CONCLUSION

We presented the data in Figures 2, 3, and 4 without error bars to assure the data points are clearly visible. The data plotted were the average value of 4 measurements and the size of the plotted points corresponding to ±0.15 mmAl of standard deviation from the average value. As indicated in Table 1, the manufacturers’ tolerance for the HVL is ±0.2 mmAl for both radiation detection systems over the HVL range of 1.3 to 13.5 mmAl (Radcal) and 1.2 to 14 mmAl (RTI), respectively. With the data and graphs presented in this manuscript, it is evident the HVL measured with SSDS included in our study are reliable instruments to evaluate if the imaging equipment is compliant with the minimum required filtration, especially, knowing the minimum HVL is normally measured from a 60 to 80 kV tube potential range. It is also clearly shown at the higher beam qualities either with higher tube potential, with the insertion of SSF or both, the measured HVL tends to be less than that measured with ionization chamber.

With respect to the “out‐of‐range” comment previously mentioned on RTI unit 1, unit 1 had been in use for 8 years while Unit #2 was purchased in 2018. Both RTI Units 1 and 2 were “out‐of‐range” at the time of measurements with the newest calibration lookup table supplied in the current software version. It so happened the same data was collected on the same angiography unit three years ago with RTI Unit #1 with the year 2017 edition of calibration lookup table or algorithm that displayed the HVL in the same SSF‐kV range at least two calibration cycles ago. Since the SSDS require energy dependence correction, further improvement is desirable to include the HVL measurements in the low kV (60 and 70) with the 0.9 mmCu SSF equipped angiography systems.

## CONFLICT OF INTEREST

There is no conflict of interest to report.

## Data Availability

The data that support the findings of this study are available from the corresponding author upon reasonable request.
